# Improving Transplant Medication Safety Through a Technology and Pharmacist Intervention (ISTEP): Protocol for a Cluster Randomized Controlled Trial

**DOI:** 10.2196/13821

**Published:** 2019-10-01

**Authors:** Casey L Hall, Cory E Fominaya, Mulugeta Gebregziabher, Sherry K Milfred-LaForest, Kelsey M Rife, David J Taber

**Affiliations:** 1 Health Equity & Rural Outreach Innovation Center Ralph H Johnson Veterans Affairs Medical Center Charleston, SC United States; 2 Department of Pharmacy Ralph H Johnson Veterans Affairs Medical Center Charleston, SC United States; 3 Department of Public Health Sciences College of Medicine Medical University of South Carolina Charleston, SC United States; 4 Department of Pharmacy VA Northeast Ohio Healthcare System Cleveland, OH United States; 5 Division of Transplant Surgery College of Medicine Medical University of South Carolina Charleston, SC United States

**Keywords:** transplant, adherence, medication adherence, medication errors

## Abstract

**Background:**

Medication errors, adverse drug events, and nonadherence lead to increased health care utilization and increased risk of adverse clinical outcomes, including graft loss, in solid organ transplant recipients. Veterans living with organ transplants represent a population that is at substantial risk for medication safety events and fragmented care coordination issues. To improve medication safety and long-term clinical outcomes in veteran transplant patients, interventions should address interorganizational system failures and provider-level and patient-level factors.

**Objective:**

This study aims to measure the clinical and economic effectiveness of a pharmacist-led, technology-enabled intervention, compared with usual care, in veteran organ transplant recipients.

**Methods:**

This is a 24-month prospective, parallel-arm, cluster-randomized, controlled multicenter study. The pharmacist-led intervention uses an innovative dashboard system to improve medication safety and health outcomes, compared with usual posttransplant care. Pharmacists at 10 study sites will be consented into this study before undergoing randomization, and 5 sites will then be randomized to each study arm. Approximately, 1600 veteran transplant patients will be included in the assessment of the primary outcome across the 10 sites.

**Results:**

This study is ongoing. Institutional review board approval was received in October 2018 and the study opened in March 2019. To date there are no findings from this study, as the delivery of the intervention is scheduled to occur over a 24-month period. The first results are expected to be submitted for publication in August 2021.

**Conclusions:**

With this report, we describe the study design, methods, and outcome measures that will be used in this ongoing clinical trial. Successful completion of the Improving Transplant Medication Safety through a Technology and Pharmacist Intervention study will provide empirical evidence of the effectiveness of a feasible and scalable technology-enabled intervention on improving medication safety and costs.

**Clinical Trial:**

ClinicalTrials.gov NCT03860818; https://clinicaltrials.gov/ct2/show/NCT03860818

**International Registered Report Identifier (IRRID):**

PRR1-10.2196/13821

## Introduction

### Background

Over the past 20 years, the use of contemporary immunosuppression has reduced the risk of rejection by more than 80%, but long-term allograft survival remains suboptimal [1,2]. Current immunosuppressants are highly effective but carry the burdens of considerable toxicities, multiple drug interactions, and complex regimens. Drug-related problems, which encompass medication errors, nonadherence, and adverse drug events, are a predominant cause of deleterious clinical outcomes in solid organ transplant recipients—most notably, graft loss [3-6]. Our previous research, as well as studies from other groups, demonstrates that drug-related problems occur in two-thirds of transplant recipients, leading to potentially avoidable hospitalizations in 1 in 8 recipients; those that develop medication errors are at considerably higher risk of graft loss [6-9].

Previous research has demonstrated that transplant recipients are burdened with numerous risk factors for the development of medication safety events. These include taking more than 10 medications concomitantly with more than 30 doses ingested per day, being prescribed narrow therapeutic index medications that are prone to drug interactions, taking chronic immunosuppressants with known debilitating side effects, and having frequent dosage adjustments. In addition, long-term ambulatory transplant recipients usually receive care across multiple health care organizations; thus, fragmented care, omissions, duplications, and discrepancies in medication regimens are common among these patients. We have also established this as a major issue facing veteran transplant recipients. Veteran transplant recipients who receive care from a transplant center outside their primary Veterans Health Administration (VHA) location are particularly at risk for these types of errors [6,7,9-11].

There is widespread consensus that the use of multiple sources of health care may hinder effective care coordination and result in care fragmentation and duplication of services, leading to poorer outcomes and higher costs [12-14]. Millions of veterans are eligible for health care services covered by their VA benefits and other insurance, such as Medicare or a private health care plan. Although the VA Health Care System offers comprehensive care, many receive health care services at both VHA and non-VHA facilities. Patients with ongoing care in both VA and non-VA settings can be described as receiving dual care and are often referred to as *dual users* [15,16]. Although the use of dual health care systems increases access and care options for veterans, dual use also increases the potential for care to be uncoordinated or fragmented[17,18]. Previous work on veterans who use both VHA and Medicare inpatient or outpatient services has found that dual (vs single) system users experienced higher rates of rehospitalization after heart failure or acute stroke and increases in mortality risk[19-22]. Among veterans with diabetes, 1 study demonstrated that dual users with diabetes were significantly more likely than VA-only users to be overtested for both hemoglobin A_1c_ and microalbuminuria, and another reported evidence of substantial overuse of glucose test strips among dual health care system users [23,24].

Veteran transplant recipients are embedded within highly complex interfacility systems of care such that medication safety monitoring and care coordination in the ambulatory care setting are often fragmented and suboptimal. Our previous research has demonstrated that nearly two-thirds of veteran transplant recipients are dual users, with 62% having multiple providers managing the same conditions. This leads to a significant number of duplications and omissions in care. Medication discrepancies between systems are nearly universal as well. Thus, provider-level and system-level issues represent substantial reinforcing and enabling factors driving medication safety events in veteran transplant recipients [11].

Early recognition of adverse drug events in transplant recipients will likely help prevent downstream clinical sequelae, including nonadherence and irreversible immunosuppressant toxicities. Research demonstrates that clinical pharmacists have the unique education and training to both identify these events early while also developing strategies to mitigate or resolve the associated sequelae [25-31].

The Improving Transplant Medication Safety through a Technology and Pharmacist Intervention (ISTEP) study seeks to improve medication safety for high-risk veterans using 2 innovative components: the utilization of a dashboard monitoring system to conduct automated surveillance for immunosuppressant safety issues and alert pharmacists when such a potential issue arises coupled with a pharmacist-led intervention to improve the management and coordination of immunosuppression therapy. The ISTEP dashboard is an expanded version of a preliminary Web-based medication safety dashboard currently used within Veterans Integrated Services Networks (VISNs) 7 and 12. Through a collaborative effort between the investigators and the Medical University of South Carolina Biomedical Informatics Center, we have expanded the dashboard to significantly improve its query and reporting capabilities. The goal of this study is to demonstrate a scalable pharmacist intervention that leverages technology and analytics to improve medication safety and clinical outcomes as well as reduced utilization at lower costs for veteran organ transplant recipients.

### Objectives

The complexities and toxicities associated with immunosuppressive medication regimens and fragmentation of care across multiple health organizations place veteran organ transplant recipients at high risk of developing medication safety issues, which can lead to hospitalization, increased health care expenditures, and ultimately graft loss. Supported by previous research [32,33], the use of a technology-enabled, pharmacist-led intervention provides a promising and innovative approach to improve medication safety and reduce drug-related problems in veteran solid organ transplant recipients. 

The study will measure the clinical and economic effectiveness of a pharmacist-led intervention that uses an innovative dashboard monitoring system that alerts pharmacists when potential drug safety issues arise to improve medication safety and health outcomes, compared with usual posttransplant care.

The primary objective of the study is to measure the effectiveness of a pharmacist-led, technology-enabled intervention on reducing the rate of hospitalizations and emergency room (ER) visits in veteran organ transplant recipients, compared with usual care. Secondary objectives include measuring the effectiveness of the intervention on reducing health care costs (compared between the intervention and control groups) and assessing the functionality and efficiency of the dashboard system. The goal of this research is to demonstrate the effectiveness of a feasible and scalable technology-enabled intervention on improving medication safety and health care costs.

## Methods

### Study Design

ISTEP is a 24-month prospective, parallel-arm, cluster-randomized, controlled multicenter study involving 10 study sites (5 sites for each study arm). Across the 10 VA health care systems, approximately 1600 veterans will be evaluated between the intervention and control groups for the primary outcome. This study has been approved by the VA central institutional review board.

### Recruitment, Screening, and Enrollment Procedures

A pragmatic clinical trial, the ISTEP study aims to test the effectiveness of the intervention in routine clinical practice using broadly inclusive criteria for study participation. Veteran patients will be included in this study if they are solid organ transplant (eg, kidney, liver, pancreas, heart, or lung) recipients who have active outpatient prescriptions for immunosuppressant medications at 1 of the 10 participating VA health care organizations.

As this is a cluster-randomized study, randomization will occur at the site level rather than the patient level. The cluster-randomized design of this study was chosen for a number of important reasons. A cluster-randomized study design allows investigators to test a promising intervention against a similar control group with respect to patient constitution and time. Randomization at the patient level, as opposed to the site, would not be feasible, as there would be a high probability of cross-contamination based on the intervention proposed and the technology component, which uses site-specific population surveillance. Finally, randomization at the patient level would have required individual patient-level consent, which would have limited our ability to have adequate power to test the outcome of interest in the 3-year study period.

Informed written consent will be obtained from the participating pharmacist(s) at each study site. Following this, each site will be randomized through computerized random number generation to standard care or standard care plus the pharmacist-led, technology-enabled intervention. To ensure a roughly equal number of patients between the 2 comparison groups, randomization will be stratified by each site’s estimated number of veteran organ transplant recipients (<125 vs ≥125 active patients). After randomization, each participating pharmacist will be informed of their assigned group. Those in the intervention arm will be trained on the dashboard system’s functionality using the dashboard and delivering the intervention. Those in the usual care group will continue to provide the same level of care they are currently providing as part of their normal day-to-day activities and job functions.

### Eligibility

#### Inclusion Criteria

Veteran organ transplant recipients will be identified using International Classification of Diseases, Ninth Revision and Tenth Revision, codes from the VA electronic health record. Patients must have an active code stating they are a recipient of an organ transplant. Patients must be receiving at least one antirejection medication dispensed by the VA site. These medications include tacrolimus, cyclosporine, azathioprine, mycophenolate, sirolimus, everolimus, or belatacept.

#### Exclusion Criteria

As a pragmatic clinical trial, exclusion criteria were kept to a minimum. All veterans meeting inclusion criteria will be monitored by the dashboard and will be included in the outcomes assessment. Patients may enter or exit the study in a rolling manner, which will be accounted for during analysis.

### Intervention

The sites randomized to the intervention arm will continue to use the current standard of care procedures within their sites while also using the dashboard system daily to identify patients with potential medication safety issues. Usual care for veteran organ transplant recipients across the 10 study sites is not standardized but generally includes the following: at most sites, nurse coordinators and/or midlevel practitioners are responsible for general transplant patient oversight, including ensuring laboratory assessments are scheduled/reviewed and medication regimens are accurate and up to date. However, large patient numbers and workload constraints preclude these health care professionals from prospective detailed daily monitoring of patients. In addition, during this long-term ambulatory phase of care for transplant patients, pharmacists usually act as consultants and are only involved in direct patient care if an issue arises, and the nurse or provider engages the pharmacist for assistance. Within usual follow-up care, pharmacists do not conduct routine daily surveillance of all transplant patients.

The intervention consists of increased review of patients by a pharmacist and increased scrutiny of patients’ medication regimens and laboratory values using a dashboard surveillance system. The dashboard will be updated at approximately 7 am every day. Participating pharmacists randomized to the intervention arm will be expected to check the dashboard for alerts at a minimum of 3 days per week. The 4 primary medication safety issues the intervention pharmacists will be alerted to and address are laboratory abnormalities, medication nonadherence, drug interactions, and medication coordination or communication issues. For each of the laboratory values that will be monitored and reported in the dashboard, a detailed algorithm will be provided delineating how to address the issue.

**Figure 1:**
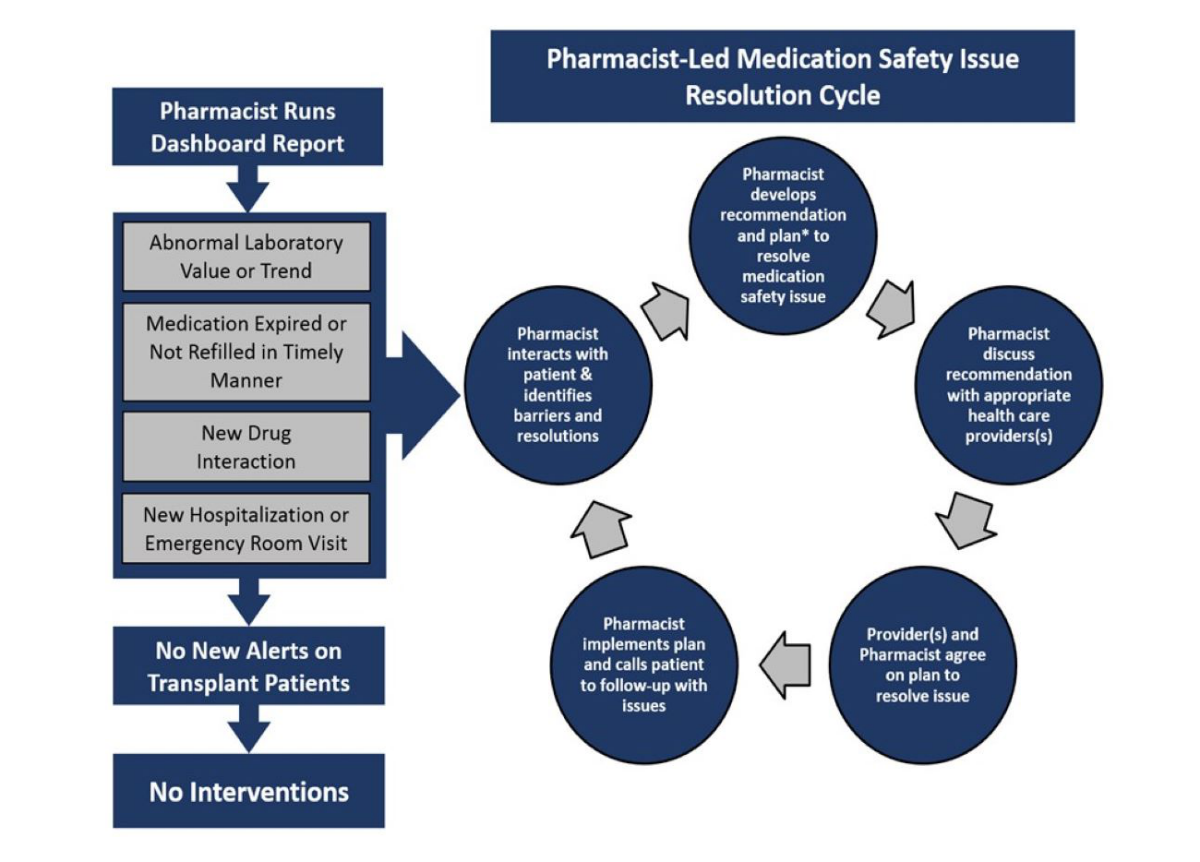
Schematic representation of the process the pharmacist will use to identify, manage, and resolve potential medication safety issues.

The pharmacist will serve as a patient navigator, intervening to resolve the medication safety issue. Once the pharmacist validates that the dashboard alert is a relevant issue, they will develop a management plan using the study’s developed protocols (Figure 1). Pharmacists will then discuss the recommendations with the relevant providers (as necessary) to agree on a plan; the pharmacist will then be responsible for implementing the plan. One example alert may be for hypomagnesemia, as this is a well-known adverse effect of calcineurin inhibitors [34]. Strategies the pharmacist may implement to address this include dietary interventions and supplementation. Other relevant alerts and corresponding interventions will be addressing out of range drug levels. These interventions may include ensuring the patient is taking the correct dose, ensuring the level is a true trough value, checking for new drug interactions, and adjusting the dose when necessary. The pharmacists will be alerted when a patient fails to refill an immunosuppressant medication in a timely manner, perhaps indicating nonadherence. When medication nonadherence is identified as a potential issue, the pharmacist will then assess this information and call the patient to address this issue. Strategies to address deliberate nonadherence include removing perceived or actual barriers, using motivational interviewing, and addressing side effects and cost concerns. For unintentional nonadherence, pharmacists can implement trigger reminder strategies, simplify regimens, and re-educate. Within drug interaction alerts, the intervention will focus on reducing the impact of these through changing of regimens (when appropriate), educating patients or providers, and increased monitoring and surveillance [35]. Alerts for medication coordination issues will encompass discharges from the ER/hospital and missed laboratory assessments. The intervention pharmacist will ensure accurate and safe medication regimens through medication reconciliation and improved medication safety surveillance through the scheduling and follow-up of laboratory assessments [26].

#### Dashboard System Specifications

The technology component of this intervention consists of the use of a dashboard system that performs population-level surveillance of organ transplant recipients and identifies those with potential drug-related problems. The dashboard system was built using Microsoft Structured Query Language (SQL) Server Management Studio, using the Python programming language and a local Web browser for the user interface. We have expanded the preliminary dashboard currently in use by VA VISNs 7 and 12 to improve its query and reporting capabilities. The SQL queries to identify the triggers required in the expanded application are stored in the Field Reporting Enclave environment. The dashboard expansion will contain 4 additional immunosuppressant safety monitoring domains.

First, the system monitors for immunosuppressant nonadherence through the tracking of refill activity and expiring medications.

The dashboard will alert if a medication has not been refilled or if a medication has expired. Pharmacists will be alerted when the patient’s proportion of days covered (PDC) for an immunosuppressant medication falls below 80%, meaning that they have had less than 80% of days’ supply on hand over the past year, as calculated by quantity and refill dates. The dashboard also includes expanded laboratory monitoring capabilities to include monitoring for missing laboratories and laboratory values (not checked in 6-12 months, depending on the laboratory value and the immunosuppression regimen) that would indicate the development of a potential adverse drug event (immunosuppressant toxicity or ineffectiveness). The dashboard will also monitor laboratory value trajectories and alert the pharmacist of patients who have trends in key laboratory values suggesting the development of a potential adverse drug event; for example, setting a trigger such that the serum creatinine concentration has increased more than 30% since the last recorded value. Finally, the dashboard will monitor drug interactions, including newly added or discontinued drugs or changes in the dose or dosing regimen of an interacting drug would also trigger an alert (Tables 1 and 2). Validation of the dashboard was conducted before the study opening and will continue during the initial deployment by the participating pharmacists at the 5 intervention sites. If there is strong evidence to suggest that components of the dashboard are not providing clinically relevant alerts or if the ratio of alerts to actionable alerts is exceedingly high, the investigational team may decide to modify this component of the system. These process measures will serve to assess the dashboard functionality and allow for modification to improve the efficiency of the intervention.

**Table 1:** Specifications of the transplant medication monitoring dashboard system–laboratory thresholds and specific drug interaction that warrant review.

Monitoring variables: laboratory assessments	Absolute value thresholds	Trajectory threshold
Potassium	<3 or >5.5 mEq/L	>30% change
Bicarbonate	<18 or >30 mEq/L	>30% change
Blood urea nitrogen	>40 mg/dL	>30% increase
Creatinine	>2.5 mg/dL	>20% change
Glucose	<60 or >200 mg/dL	>30% change
Calcium	<7 or >10 mg/dL	>30% change
Magnesium	<1.0 or >2.5 mEq/L	>30% change
Phosphorus	<2.0 or >5.0 mg/dL	>30% change
White blood cell count	<3.0 or >15.0 cells/mm^3^	>30% change
Hemoglobin	<8 or >15 gm/dL	>20% change
Platelets	<50 or >500 cells/mm^3^	>30% change
Aspartate aminotransferase	>60 U/L	>20% increase
Total bilirubin	>1.5 mg/dL	>20% increase
Hemoglobin A_1c_	>8%	>20% increase
Low-density lipoprotein	>130 mg/dL	>20% increase
Triglycerides	>300 mg/dL	>20% increase
Tacrolimus trough	<3 or >15 ng/mL	>20% change
Cyclosporine trough	<30 or >400 ng/mL	>20% change
Sirolimus trough	<2 or >8 ng/mL	>20% change
Everolimus trough	<2 or >8 ng/mL	>20% change

**Table 2:** Specifications of the transplant medication monitoring dashboard system–specific drug interaction that warrant review.

Interacting drugs		Trigger definitions
**Enzyme inhibitors**		
	Macrolides (clarithromycin, erythromycin, telithromycin); Azoles (ketoconazole, itraconazole, voriconazole, posaconazole, fluconazole)	Initiation, discontinuation, and dose change >20%
	Calcium channel blockers (diltiazem, verapamil)	Initiation, discontinuation, and dose change >20%
	HIV (nafazodone, delaviridine, saquinavir, nelfinavir, indinavir, amprenavir)	Initiation, discontinuation, and dose change >20%
	Miscellaneous (boceprevir, telaprevir, cimetidine, chloramphenicol, danazol)	Initiation, discontinuation, and dose change >20%
**Enzyme inducers**		
	Antiepileptics (carbamazepine, phenytoin, phenobarbital)	Initiation, discontinuation, and dose change >20%

### Data Collection

Overall, 2 general types of data will be collected: (1) operational data used for the dashboard system and (2) research data to assess the impact of the intervention on outcomes. Operational data for the dashboard will be captured through querying the national VA Corporate Data Warehouse (CDW) operational database. These data elements include diagnoses, laboratory values, medication regimens, refill histories, provider types, and health care encounters (hospitalizations and ER visits), gathered and queried daily. The research CDW system (VA Informatics and Computing Infrastructure [VINCI]) will provide data to assess outcomes occurring within the VA health care system, including hospitalizations, ER visits and costs, and mortality. CDW data will also be used to assess interventions by querying pharmacists’ progress notes. To ensure encounters are captured in a comprehensive manner, we will also link the VA CDW data to Centers for Medicare and Medicaid Services (CMS) claims data and capture non-VA ER and hospitalization encounters (after study completion). The CMS data will provide non-VA health care utilization, including hospitalizations and ER visits, as well as non-VA cost estimates. Scientific Registry of Transplant Recipients data will provide all baseline donor, recipient, and transplant characteristics and clinical outcomes, including acute allograft rejection, graft loss, and death. Queries answered by intervention pharmacists will include the number of alerts received, how many were considered clinically relevant/actionable, time to conduct the intervention, and general intervention types.

### Outcome Measures

The outcomes to be assessed for this study all relate to evaluating the impact of the intervention. The primary outcome measure for this study will include the overall rate of ER visits and hospitalizations, compared between the intervention and control groups, while adjusting for baseline patient, provider, and facility characteristics. As previously stated, we will link the VA CDW data to the CMS (Medicare) claims data to capture both VA and non-VA ER and hospitalization encounters and provide a more accurate assessment of health care utilization. ER visits and hospitalizations will be assessed and compared as described in the section Sample Size and Statistical Analysis Plan.

Secondary outcomes to be assessed include a cost-benefit analysis of health care costs between the intervention and control groups as well as an assessment of the dashboard system’s functionality and efficacy. Overall health care costs accrued during the 24-month study and those accrued in the 24 months before study initiation will be analyzed and compared between the control and intervention groups. Cost data will be standardized using the VA Health Economics Resource Center definitions, which normalize regional differences in costs because of variation in cost of living indices. As with the primary outcome, we will also acquire and link CMS claims data to gain a comprehensive assessment of costs, including those that accrue from non-VA care (after study completion). Another secondary outcome is to assess the success of the dashboard systems expansions and utilization. To do so, we will evaluate the dashboard’s functionality by measuring and reporting descriptive statistics for the alert numbers, alert relevance, time, and the actions taken with regards to the alert and the intervention magnitude. Alert and intervention information will be entered by intervention pharmacists through the dashboard interface, which will then be brought into VINCI for formal analysis. These measures will allow us to ascertain if the expanded dashboard is meeting expectations, with regards to functionality and efficiency. We will also assess the number of potential immunosuppression safety issues that occur and compare these between the 2 study arms. To do so, we will use the dashboard to provide monthly measurements of the following: percentage of patients with missing laboratory assessments; percentage of patients with alarming laboratory values without follow-up scheduled; and mean adherence to immunosuppression, based on refill timeliness and estimated using the PDC; percentage of patients with a significant drug interaction without an immunosuppressant level; and percentage of patients with hospital discharge or ED visit without follow-up scheduled. These will be measured in all patients and compared between the intervention and control groups at monthly intervals. 

### Sample Size and Statistical Analysis Plan

On the basis of the projected enrollment numbers, we expect to have ample power to detect a statistically significant difference between the intervention and control groups with regards to the primary aim of hospitalization and ER visit rates. We used data from a recent national study conducted between 2009 and 2012 [36]. These results demonstrated that the rate of ER visits after transplant was 1.27 per person-year, and 48% of those ER visits resulted in hospitalization. We used a conservative estimate of an intracluster correlation of 0.05 and calculated a sample size of 1350 participants to allow us to detect a 25% relative decrease in ER visit rate and hospitalization rate with 80% power. The 25% relative improvement in rates is a conservative estimate of intervention effect, based on previous pharmacist-led initiatives the investigators have conducted [25,37]. After allowing for 15% loss to follow-up, we need a total of 1600 participants to achieve the study goals. We will stratify site randomization by estimated site sample size (<125 vs ≥125) to ensure an approximate even distribution of patients across study arms. These power calculations were conducted using a 2-sided test for counts with Poisson regression adjusting for intracluster correlation and with alpha set at .05.

For comparative statistical assessments of utilization outcomes (ER visits and hospitalizations), the 2 groups will be compared using a generalized linear mixed models (GLMM) approach [38]. This approach allows for the measurement of participants at different time points, clustering by study site, missing data under the assumption of missing at random, and time-varying or invariant covariates and can also account for the effect of correlated longitudinal measurements within participants. Outcomes that are measured longitudinally, such as graft loss and mortality, will have intervention group, time, and time-by-intervention group as primary independent variables in the model. Additional adjustment covariables will be added to the model in a second set of analyses. Covariates will include patient sociodemographics: age, sex, race, comorbidities (diabetes, hypertension, and heart failure), marital status, and education.

For the cost analysis, we will also use multivariable modeling and propensity score calibration [39]. We will assess the effect of the intervention on different sources of cost at the patient level, which include inpatient, outpatient, and pharmacy in addition to the total aggregated cost. The cost models will be estimated using log-normal or gamma models (special cases of GLMM) to examine the association of the intervention with cost, adjusting for the aforementioned patient sociodemographics, donor information, and transplant characteristics. We will estimate different models adjusting for the clinical outcomes to examine the robustness of the results.

For the assessment of the functionality of the dashboard system and the time required to complete the intervention, we will use standard descriptive statistics for these measurements, including mean (SD), median (interquartile range), proportion (percentage), and 95% CI. Missing data will be handled using several techniques, including multiple imputation and maximum likelihood [40]. Missing data mechanisms will be examined using both univariate and multivariate methods.

## Results

This study is ongoing. Institutional review board approval was received in October 2018 and the study opened in March 2019. To date, there are no findings from this study, as the delivery of the intervention is scheduled to occur over a 24-month period. The first results are expected to be submitted for publication in August 2021.

## Discussion

### Overview

The use of the dashboard monitoring system to conduct automated near real-time surveillance for immunosuppressant safety issues and alert pharmacists when such issues arise is innovative in several ways. First, this technology leverages the currently underused enormity of data that are already embedded within the VA electronic health record system. Owing to the complexity involved in the clinical management of transplant recipients, there are substantial numbers of laboratory values and medication regimen alterations that occur within each patient. Automating the monitoring of these data to identify trends and potential patient issues allows for improved efficiency. Medication refill adherence and relevant drug interaction monitoring improve the comprehensive assessment of medication adherence and safety. Finally, monitoring for hospitalization and ER visits will allow for appropriate follow-up with the transplant teams when necessary. The use of a pharmacist-led intervention is also innovative. Although the use of clinical pharmacists to improve medication safety and outcomes is well documented, there are limited studies analyzing the effectiveness of such interventions within the transplant population, and none specifically within veteran organ transplant recipients. The limited studies that demonstrate improved medication outcomes using pharmacists’ led interventions among transplant patients (a number of which are from our research group) predominantly focus on the acute perioperative hospitalization phase [25,26,41-44]. The innovative component of this proposal is the use of a pharmacist-led intervention during the longitudinal ambulatory phase, following the posttransplant surgical event. As this has now been recognized as a major contributor to medication safety issues and subsequent graft loss, studies are needed that focus efforts on improving care during this period [2,45]. Pharmacists are uniquely trained to identify medication safety issues early in their course while also capable of developing and implementing strategies to mitigate or resolve these issues and assist patients transitioning from acute to chronic phases of posttransplant care [26,28,30].

There are several challenges with health services research that have the potential to undermine the intervention. First, as this intervention seeks to improve medication safety through modifying human behaviors, there is potential for implementation issues associated with the pharmacist-led intervention; there may be actions by the patient or provider that may limit or undermine the impact of the intervention. To maintain the fidelity and consistency of the intervention, we will use structured interventions based on identified barriers, develop a detailed standard operating procedure manual for the intervention, and conduct face-to-face training with the pharmacists. Systems barriers also have the potential to limit the intervention impact. As these patients are routinely managed across multiple health care systems, both inside and outside the VA, it is important that the pharmacist-led intervention facilitates medication safety and coordination across these systems. We will train the pharmacists on the best methods to ensure optimal coordination of care for these patients and provide tools that we currently use to improve the efficiency of outside care documentation.

### Conclusions

Supported by previous research, the use of a technology-enabled, pharmacist-led intervention provides a promising and innovative approach to improve medication safety and reduce drug-related problems in veteran organ transplant recipients. Successful completion of the ISTEP study will provide empirical evidence of the effectiveness of a feasible and scalable technology-enabled intervention on improving medication safety and costs. We envision this technology can be used in the monitoring of all US transplant patients receiving care within the VA. Our long-term goal is to leverage the use of this technology to develop a VA-specific pharmacist learning collaborative to substantially improve immunosuppressant safety and outcomes within veteran organ transplant recipients.

## Appendix

Multimedia Appendix 1 Peer-reviewer report from Health Services Research and Development.

## Abbreviations

CDW: Corporate Data Warehouse
CMS: Centers for Medicare and Medicaid Services
ER: emergency room
GLMM: generalized linear mixed models
ISTEP: Improving Transplant Medication Safety through a Technology and Pharmacist Intervention
PDC: proportion of days covered
SQL: Structured Query Language
VA: Veterans Administration
VHA: Veterans Health Administration
VINCI: Veterans Administration Informatics and Computing Infrastructure
VISN: Veterans Integrated Services Network
