# Supplementary material for: Improving Transplant Medication Safety Through a Technology and Pharmacist Intervention (ISTEP): Protocol for a Cluster Randomized Controlled Trial
Source: JMIR Res Protoc. 2019 Oct 1;8(10):e13821. doi: 10.2196/13821 (PMC6774238; doi:10.2196/13821)
Supplement: Multimedia Appendix 1 [file resprot_v8i10e13821_app1.pdf]

SUMMARY STATEMENT

PROGRAM CONTACT: **(Privileged Communication)** ***Release Date:*** **04/14/2017**

Revised Date:

Application Number: **1 I01 HX002130-01A2**

Principal Investigator

TABER, DAVID J.

Applicant Organization: RALPH H JOHNSON VA MEDICAL CENTER

Review Group: **HSR1**
HSR-1 Medical Care and Clinical Management; Health Professional Behavior

Meeting Date: **03/07/2017** ***RFA/PA:*** **HX17-001**
Council: **MAY 2017**
Requested Start: **07/01/2017**

Project Title: **Improving Transplant Medication Safety through a TEchnology and Pharmacist (ISTEP) Intervention in Veterans**
SRG Action: **Impact Score:167 Percentile:4.9**

Human Subjects: **20-Human subjects involved - No exemption designated**
Animal Subjects: **10-No live vertebrate animals involved for competing appl.**

Project Year	Direct Costs Requested
1	238,607
2	230,872
3	233,150
4	239,528
TOTAL	942,157

KEY SUMMARY POINTS:

1. The study addresses a high cost, fragile health population with a potentially important intervention.

2. Motivational interviewing and self-management support skills aimed at improving adherence require substantial training that does not appear to be sufficiently included in the study (see Reviewer 2).

3. Nonadherence is broadly defined based on receipt of prescription and not actually taking the medication as prescribed.

DESCRIPTION (provided by applicant):

Anticipated Impacts on Veterans Health Care: The first three essential strategies listed within the VA's Blueprint for Excellence encompass plans to improve care to vulnerable Veterans, deliver high quality care through achieving the “Triple Aim” and leverage the use of technology to improve the efficiency of care delivery. The intervention this grant proposes focuses on improving medication safety and care coordination within a high risk vulnerable Veteran population, leverages the use of informatics and analytics to support this intervention, and aims to demonstrate improved care at reduced costs through the pharmacist intervention; thus, perfectly aligning with these three essential components of the Blueprint. The overarching goal of this study is to develop a feasibly deployable, technology-enabled intervention that will demonstrate substantial improvements in immunosuppressant medication safety, clinical outcomes and health care costs in Veteran organ transplant recipients; demonstrating this through a randomized controlled trial will provide sufficient evidence to further develop a VA-specific pharmacist learning collaborative aimed at improving care and reducing costs for Veteran organ transplant recipients across the entire VA system.

Background: Organ transplant is the gold-standard treatment for patients with end organ diseases of the kidney, liver, heart and lungs, as it substantially improves survival and quality of life. Over the past 20 years, the use of contemporary immunosuppression has reduced the risk of acute rejection rates by upwards of 80%; yet long-term allograft survival remains suboptimal. Studies have demonstrated that causes of late graft loss is predominantly driven by immunosuppression adverse events and late allograft rejection episodes from medication errors and non-adherence, which encompass issues directly related to medication safety. Our research demonstrates that medication errors occur in nearly two-thirds of transplant recipients, leading to hospitalization in 1 in 8 recipients. Recipients that develop significant medication errors are at considerably higher risk of graft loss, leading to higher costs and mortality. Thus, in order to improve medication safety and long-term outcomes in transplant recipients, enhancements in immunosuppressant therapy management is needed.

Objectives: The central hypothesis for the ISTEP study (Improving Transplant Medication Safety through a TEchnology and Pharmacist Intervention) is that pharmacist-led immunosuppressant therapy management, facilitated through the use of innovative technology, will significantly improve immunosuppressant safety and clinical outcomes in Veteran transplant recipients.

Methods: This is a 24-month, prospective, multicenter, cluster-randomized controlled clinical trial at 10 sites, randomizing 5 sites to standard clinical care and 5 to standard care and the technology-enabled pharmacist intervention. The technology component of this intervention consists of the use of an expanded dashboard system that has already demonstrated effectiveness in improving immunosuppression monitoring. The dashboard performs population-level surveillance of transplant recipients and identifies those with potential drug-related problems, including non-adherence, drug interactions, missing and worrisome trends in labs; then providing a real-time alert to the pharmacist, who will determine its relevance and intervene in an appropriate protocol-guided manner. Effectiveness will be determined by comparing the rates of hospitalizations and ER visits between groups, while

adjusting for baseline patient, provider and facility characteristics. Secondary measures include comparing healthcare costs and determining dashboard functionality, dashboard actionability and pharmacist intervention types and acceptance rates. We will also assess the overall incidence and severity of drug-related problems and graft and patient survival rates and compare these between the intervention and control sites.

CRITIQUE 1

1. Significance.

The investigators make a strong case for the needed improvement in care for 12,000 patients receiving immunosuppression. Their prior work shows significant medication “issues” and the high costs and health concerns associated with these issues. The 10 study sites treated 1,689 patients, roughly 14% of the target population (7% in the study group); if this project works, they will have made significant headway in providing appropriate care to affected patients.

2. Approach.

This two-part study seeks to 1) refine the dashboard tool and train the personnel; 2) evaluate the utility of the tool (Aim 3); and 3) assess the consequences of its use (Aim 1) and its cost-effectiveness (Aim 3).

The investigators propose a randomized design for five study sites and five control sites led by pharmacists to follow patients for two years, with a final year for analyses and dissemination of results – including planning for broader implementation. The approach towards development and training is reasonable. The randomized study is important, recognizing that n=10 and n=1,689; this is both a test of whether pharmacists will use the dashboard as it is whether use of the dashboard and the pharmacists’ subsequent intervention makes a difference in use of hospital and emergency department (ED) services.

The cost-benefit analysis will have all investment costs (first year) and operational costs and be limited in benefits to two years, with benefits expected only in the second year of intervention (third study year). Analysis plan for count data of costs (hospitalizations and ED visits) is complete. Analysis plan for cost data include propensity scoring (with unstated criteria) and multivariate analyses that include socio-demographic, donor and transplant characteristics. It is acknowledged that sample size calculations for counts are not likely to hold for cost data.

3. Innovation.

The innovation is a new dashboard that identifies key medication measures and threshold values (or trend values) for these measures to lead to alerts. Presumably, there is reasonable science behind the expert assessment of values leading to alerts so the development process will be one of compiling information and not requiring new research. The prior development of a laboratory tests dashboard indicates the investigators’ skills and awareness of structuring dashboards.

They propose critical evaluation of the dashboard – something that is not often done, but clarity on how this evaluation will be conducted is needed. The “Overhage” method was developed in 1999 and has been cited 103 times, with an unknown number of uses in practice. Its aim is to assess good use of pharmacists' time and activities. This is slightly different from an engineering evaluation of false positives/negatives, timeliness, appropriateness of the threshold and trend values, etc. Still, the

evaluation is important to the overall study as the appropriate use of pharmacists' time is a key cost driver to the study. There are many evaluations of computer prompts in healthcare that were not cited.

Some of the dashboard elements are not innovative. Every pharmacy has a set of drug interaction alerts. The innovation is the use of threshold / trend values and whatever intuition or science has been applied to interactions that arise for combinations or dose-dependent concerns.

The pharmacist intervention – acting on the dashboard may or may not be innovative. Abnormal values, drug interactions and medical visits lead to deliberate actions. Expired or lack of refills is a starting point for identifying adherence issues which appear to account for a substantial portion of adverse events, require pharmacists' "social" interventions. The social interventions include motivational interviewing and other educational tools that will be providing at a training session. The evidence is weak on the effectiveness of social interventions by medical professionals and not well presented in the submission.

4. Investigator Qualifications; Facilities and Resources.

These are highly qualified investigators with the appropriate facilities and resources. The team is possibly missing a trainer for social interventions. The investigators have no reported record of training on social interventions and have no experts in social work/psychology/education for support.

5. Multiple PI Leadership Plan.

Not applicable.

6. Adequacy of Response to Previous Feedback Provided by HSR&D Regarding the Proposed Study.

The prior feedback was very detailed and the investigators provided point-by-point responses that address the feedback.

7. Protection of Human Subjects from Research Risk.

The protection of human subjects appears to be fine.

8. Inclusion of Women and Minorities in Research.

The inclusion of women and minorities is fine and consistent with the study population.

9. Budget.

The budget is fine and consistent with study activity, and much early work has made the dashboard development feasible.

10. Data Management and Access Plan (DMAP).

The investigators have demonstrated their ability to collect CMS data for these Medicare eligible patients (qualified by ESRD) and VA data. Collection of pharmacists' actions, efforts, and time will be input into CPRS and a REDCap hosted tool. There are challenges associated with reporting that appear to have been reasonably anticipated by the investigators.

11. Overall Impression.

The investigators seek to address medication issues that are an important concern for patients on immunosuppression. A dashboard that highlights issues and directs pharmacist intervention – in an environment where pharmacists can intervene – is a seemingly good tool. The medication and/or system related issues resulting in alerts can be directly addressed by the pharmacist. The non-adherence (on refills, an indirect measure of medication use) issues require pharmacists' use of social interventions, for which the evidence is weak and the investigators may not be fully prepared to deliver. The analysis plan is reasonable, though propensity scoring and multivariate analysis plans are indicated, but not specified. If all goes well, the investigators have provided for good dissemination of the tools.

12. Key Strengths.

1. The study addresses a high cost, fragile health population, potentially aided in a meaningful way by a new-age intervention.

2. Electronic medical records-based alerts are increasingly common and lessons on use could benefit efforts beyond the study population.

3. Pharmacists in the VA are in a position to undertake direct actions and communications based on alerts – reducing the complexity of the care process for these patients.

4. A good extension of the work plan of the investigators.

5. Prior work is suggestive that Aim 1 will show significant results and Aims 2 and 3 will show suggestive results.

13. Key Weaknesses.

1. Dashboards are increasing in frequency and complexity, perhaps leading to alert burnout, though they are taking many efforts to minimize pharmacist burden.

2. Motivational interviewing and educational efforts require substantial training that does not appear to be in the skill sets of the investigations, and exhibit low fidelity in practice even among programs provided by skilled trainers.

3. The cost-benefit analysis will likely only be suggestive, as the variations in costs will likely be very large relative to the sample size. Analysis plan for cost data is not well specified.

CRITIQUE 2

1. Significance.

This proposal builds an extremely strong argument for the importance of addressing medication safety issues among the 12,000 transplant recipients receiving care in the VA with immunosuppressant medication. The potential for both death and other negative clinical outcomes and increased healthcare utilization is significant. Much of the evidence around this topic in the VA comes from these investigators. Further, the proposal recognizes that efforts to address the medication related issues facing transplant patients require a multi-component process. In this case, they are testing having a technology innovation that alerts the pharmacists to potential problems and enhances the clinical pharmacist role and the availability of expert consultation.

2. Approach.

This proposal builds an extremely strong argument for the importance of addressing medication safety issues among the 12,000 transplant recipients receiving care in the VA with immunosuppressant medication. The potential for both death and other negative clinical outcomes and increased healthcare utilization is significant. Much of the evidence around this topic in the VA comes from these investigators. This proposal also builds on important evidence for the role of clinical pharmacists and the need to have real-time electronic monitoring of safety issues to aid the role of the pharmacist.

There are important positive aspects to the study design that came out of the previous review.

- The parallel cluster-randomized trial across 10 sites is very appropriate.
- The sites have been specifically identified and heavily involved in planning the grant.
- While the process of making change to the dashboard is very ambitious, the proposal includes a strong argument that the team can accomplish this effort because they are making changes to an already used dashboard and that the investigative team has the ability to quickly agree upon changes.
- The application specifically indicates the differences between the intervention and control arms. The intervention includes three important components: 1) the dashboard; 2) enhances clinical pharmacist role; and 3) availability of expert consultation. It also recognizes that patient adherence and access to the services (e.g., nutrition) needs to be addressed.
- The proposal specifically addresses the extensive training needs of the involved pharmacist.
- Outcomes are quite appropriate. The utilization outcomes are appropriate and quite important. The application includes a strong argument that these should be primary outcomes and that clinical endpoint assessment should be exploratory.
- The analysis section is well written.

There are a couple of issues not really addressed that the investigators that would need to be considered as the application processes are finalized.

- The training session does not indicate that the pharmacists will be trained on the process of addressing patient adherence. It is not clear if the pharmacist will do this with individual patients or if they will provide referrals for this process.
- It is very interesting that this proposal can do a true cost-benefit analysis. While the patient care costs are well described, the process of measuring intervention costs is not described.

3. Innovation.

The investigators recognize that efforts to address the medication related issues facing transplant patients require a multi-component process. In this case, they are testing having a technology innovation that alerts the pharmacists to potential problems, enhance clinical pharmacist role, and availability of expert consultation. The dashboard goes beyond something that could be used for quality

improvement to a tool focused on helping with real time clinical decision making. It is also quite helpful that Aim 3 will focus on the usability and feasibility of the process, including whether the dashboard is providing clinically relevant information.

4. Investigator Qualifications; Facilities and Resources.

This is a highly qualified team that covers the range of expertise needed to successfully complete this research.

5. Multiple PI Leadership Plan.

Not applicable.

6. Adequacy of Response to Previous Feedback Provided by HSR&D Regarding the Proposed Study.

The investigators continue to be exceptionally responsive to reviewer feedback. They specifically address all of the questions and made substantial changes to the proposal that make is easy to follow and more methodologically strong.

7. Protection of Human Subjects from Research Risk.

No concerns regarding protection of human subjects.

8. Inclusion of Women and Minorities in Research.

No concerns regarding inclusion of women and minorities.

9. Budget.

No budgetary concerns.

10. Data Management and Access Plan (DMAP).

No concerns with the data management and access plan.

11. Overall Impression.

The investigators were extremely responsive to reviewer feedback. This is now a very well written and methodologically strong application. There is explicit recognition of the importance of having a three competent intervention (dashboard + enhanced pharmacist role + availability of expert consultation). Outcomes are appropriate. It is helpful that the investigators will specifically look at both cost-benefit, because there is opportunity for the intervention to be cost savings, and clinical appropriateness and feasibility of the intervention.

12. Key Strengths.

1. Important topic area addressed with a multi-component intervention.

2. This is a very strong team.

3. Sites are identified and highly engaged.

4. Appropriate outcomes and well written analysis plan is presented.

5. Project is specifically evaluating clinical appropriateness and feasibility of the intervention (Aim 3).

13. Key Weaknesses.

While not really a major weakness, the investigators need to explicitly consider the pharmacists role in training related to encouraging patient medication adherence.

CRITIQUE 3

1. Significance.

These investigators are attempting to use a pharmacist driven intervention with a dashboard to reduce medication errors in VA patients who have had transplants. The hypothesis is that this intervention will improve medication safety, clinical outcomes, and reduce health care costs.

2. Approach.

The investigators propose a 24-month prospective, multicenter cluster randomized-controlled clinical trial at 10 sites, five receiving standard care and five receiving the intervention. The dashboard system is already in place. The pharmacist will review the dashboard in real time to identify and act upon medication related problems. The rates of hospitalization and ED visits will be compared, adjusting for baseline patient, provider, and facility characteristics. Intermediary process measures include dashboard functionality and actionability, pharmacist intervention types and acceptance rate, as well as overall incidence of drug-related problems.

3. Innovation.

Using pharmacist for direct intervention around drug issues is innovative.

4. Investigator Qualifications; Facilities and Resources.

Investigators are qualified to perform the work.

5. Multiple PI Leadership Plan.

Not applicable.

6. Adequacy of Response to Previous Feedback Provided by HSR&D Regarding the Proposed Study.

The investigators have made a concerted effort to respond to the previous critiques and have substantially revised the methods and analysis sections in particular.

7. Protection of Human Subjects from Research Risk.

The protection of human subjects is adequate.

8. Inclusion of Women and Minorities in Research.

The inclusion of women and minorities is adequate.

9. Budget.

Appropriate budget for proposed work.

10. Data Management and Access Plan (DMAP).

Appropriate data management and access plan for proposed work.

11. Overall Impression.

The investigators have done a very good job in revising this proposal based on the critiques of the previous reviewers. The idea of using a pharmacist to have direct contact and carry through interventions based on a dashboard is innovative, and has the potential to significantly improve outcomes as well as to decrease costs. The work done in this study could be used as a template for similar innovations serving other patient populations with complicated drug regimens.

12. Key Strengths.

1. Impact on pharmacist workload has been investigated in depth and the hope is that the intervention will positively impact efficiency.

2. The investigators have addressed how covariates will be specified and measured.

3. How data will be handled in Redcap regarding number of alerts as well as actions on alerts is more clearly specified.

4. The methods and analysis sections in general are significantly improved and address queries raised by previous reviewers.

13. Key Weaknesses.

1. Hospitalizations and ED visits are process measures and not clinical outcomes. It is unclear if just comparing numbers in this regard is adequately reflective of impact of drug medication unless there is specific detail on the reasons for these hospitalizations and visits.

2. Patient compliance is likely responsible for a significant percentage of medication errors and it is not clear how this intervention would reduce this. The dashboard does measure refill data. What exactly is this percentage in their pre-data and how would the intervention address this?

MEETING ROSTER
HSR-1 Medical Care and Clinical Management; Health Professional Behavior
Health Services Research Parent IRG
Office of Research & Development

HSR1
03/07/2017

CHAIRPERSON(S)

BERNSTEIN, STEVEN J. MPH, MD
RESEARCH SCIENTIST
ANN ARBOR VA HSRD CENTER OF INNOVATION
PROFESSOR
DEPARTMENT OF MEDICINE
UNIVERSITY OF MICHIGAN
ANN ARBOR, MI 48109

MEMBERS

ARON, DAVID C., MD, MS
STAFF PHYSICIAN
CLEVELAND VA MEDICAL CENTER
PROFESSOR
SCHOOL OF MEDICINE
CASE WESTERN RESERVE UNIVERSITY
CLEVELAND, OH 44106

ASHRANI, ANEEL ARJUN, MD, MS *
ASSISTANT PROFESSOR
MAYO CLINIC
HEMATOLOGY
ROCHESTER, MN 55905

BADER, ANGELA MPH MD *
PROFESSOR OF ANAESTHESIA
HARVARD MEDICAL SCHOOL
VICE CHAIR, PERIOPERATIVE MEDICINE
BRIGHAM & WOMEN'S HOSPITAL
BOSTON, MA 02115

BOOCKVAR, KENNETH S., MD, MS *
ASSOCIATE DIRECTOR
GERIATRICS RESEARCH, EDUCATION, AND CLINICAL CTR.
JAMES J. PETERS VAMC
PROFESSOR
SCHOOL OF MEDICINE, MOUNT SINAI
NEW YORK, NY 10029

CHEN, YUYING, PHD, MD *
ASSOCIATE PROFESSOR
NATIONAL SPINAL CORD INJURY STATISTICAL CENTER
UNIVERSITY OF ALABAMA AT BIRMINGHAM
BIRMINGHAM, AL 35294

CHUANG, EMMELINE, PHD *
ASSISTANT PROFESSOR
DEPARTMENT OF HEALTH POLICY AND MANAGEMENT
UNIVERSITY OF CALIFORNIA SCHOOL OF PUBLIC HEALTH
LOS ANGELES, CA 90095

FU, STEVEN MSCE, MD *
CORE INVESTIGATOR
MINNEAPOLIS VA HEALTH CARE SYSTEM
DIRECTOR AND ASSOCIATE PROFESSOR
UNIVERSITY OF MINNESOTA MEDICAL SCHOOL
MINNEAPOLIS, MN 55417

GATTONI-CELLI, SEBASTIANO, MD *
RESEARCH HEALTH SCIENTIST
RALPH H. JOHNSON VAMC
PROFESSOR
DEPARTMENT OF RADIATION ONCOLOGY
MEDICAL UNIVERSITY OF SOUTH CAROLINA
CHARLESTON, SC 29403

GIFFORD, ALLEN L, MD *
STAFF PHYSICIAN AND LEAD CLINICIAN
EDITH NOURSE ROGERS MEMORIAL VETERANS HOSPITAL
PROFESSOR
DEPARTMENTS OF PUBLIC HEALTH AND MEDICINE
BOSTON UNIVERSITY
BEDFORD, MA 01730

GOLDSTEIN, MARY K. MS, MD *
CHIEF
GERIATRIC RESEARCH, EDUCATION & CLINICAL CENTER
VA PALO ALTO HEALTH CARE SYSTEM
PROFESSOR
STANFORD UNIVERSITY SCHOOL OF MEDICINE
PALO ALTO, CA 94304

GOLDSTEIN, ROBERT E., MD *
PROFESSOR
DIVISION OF CARDIOLOGY
UNIFORMED SERVICES UNIV OF THE HEALTH SCIENCES
BETHESDA, MD 20814

HIGGINS, PATRICIA A., PHD, RN, BSN *
SENIOR RESEARCHER
GRECC VAMC
ASSOCIATE PROFESSOR
CASE WESTERN RESERVE UNIVERSITY
CLEVELAND, OH 44106

HUANG, SEAN SHENGHSIU, PHD *
ASSISTANT PROFESSOR
GEORGETOWN UNIVERSITY
DEPARTMENT OF HEALTH SYSTEMS ADMINISTRATION
WASHINGTON, DC 20057

JACKSON, GEORGE, PHD L. MHA, PHD *
RESEARCH HEALTH SCIENTIST
DURHAM VAMC
ASSOCIATE PROFESSOR
DUKE UNIVERSITY AND UNIVERSITY OF NORTH CAROLINA
CHAPEL HILL
DURHAM, NC 27705

KESTER, KENT E., MD *
VICE PRESIDENT & HEAD
TRANSLATIONAL SCIENCE AND BIOMARKERS
SANOFI PASTEUR, INC.
SWIFTWATER, PA 18370

LUND, BRIAN, PHMD *
CORE INVESTIGATOR
IOWA CITY VA MEDICAL CENTER
ADJUNCT ASSISTANT PROFESSOR
DEPARTMENT OF EPIDEMIOLOGY
UNIVERSITY OF IOWA COLLEGE OF PUBLIC HEALTH
IOWA CITY, IA 52246

MALLINSON, TRUDY RUTH, PHD *
ASSOCIATE PROFESSOR
DEPARTMENT OF CLINICAL RESEARCH AND LEADERSHIP
OFFICE FOR CLINICAL PRACTICE INNOVATION
THE GEORGE WASHINGTON UNIVERSITY
WASHINGTON, DC 20007

MCDONOUGH, CHRISTINE M, PHD *
RESEARCH ASSISTANT PROFESSOR
HEALTH & DISABILITY RESEARCH INSTITUTE
BOSTON UNIVERSITY SCHOOL OF PUBLIC HEALTH
BOSTON, MA 02118

NEY, JOHN, MD *
NEUROLOGIST
BEDFORD VA MEDICAL CENTER
ASSISTANT PROFESSOR
BOSTON UNIVERSITY
BEDFORD, MA 01730

PUGH, MARY JO, PHD *
RESEARCH SCIENTIST
SOUTH TEXAS VAHCS
ASSOCIATE PROFESSOR
DEPT. OF EPIDEMIOLOGY AND BIOSTATISTICS
UNIVERSITY OF TEXAS HEALTH SCIENCE CENTER
SAN ANTONIO, TX 78229

SANKARI, ABDULGHANI PHD, MD *
ASSOCIATE PROGRAM DIRECTOR
JOHN D. DINGELL VAMC
ASSOCIATE PROFESSOR OF MEDICINE
WAYNE STATE UNIVERSITY
DETROIT, MI 48201

SAYERS, STEVEN L., PHD
CLINICAL PSYCHOLOGIST, DIRECTOR OF ADVANCED
FELLOWSHIP PROGRAM IN MENTAL ILLNESS RESEARCH
PHILADELPHIA VA MEDICAL CENTER
ASSOCIATE PROFESSOR OF PSYCHOLOGY
UNIVERSITY OF PENNSYLVANIA SCHOOL OF MEDICINE
PHILADELPHIA, PA 19104

SMITH, DEAN G., PHD *
DEAN AND PROFESSOR
SCHOOL OF PUBLIC HEALTH
LSU HEALTH SCIENCES CENTER – NEW ORLEANS
NEW ORLEANS, LA 70112

STARMER, HEATHER MA CCC SLP BCS-S *
CLINICAL ASSISTANT PROFESSOR
DEPTARTMENT OF OTOLARYNGOLOGY
DIRECTOR
HEAD AND NECK CANCER SPEECH AND SWALLOWING
STANFORD UNIVERSITY
STANFORD, CA 94305

UPHOLD, CONSTANCE R., PHD *
ASSOC DIRECTOR, IMPLEMENTATION & OUTCOMES RES
GERIATRIC RESEARCH EDUCATION CLINICAL CENTER
GAINESVILLE VA MEDICAL CENTER
ASSOCIATE PROFESSOR OF AGING & GERIATRICS
UNIVERSITY OF FLORIDA COLLEGE OF MEDICINE
GAINESVILLE, FL 32608

WILKINS, EDWIN G, MD *
PROFESSOR OF SURGERY
SECTION OF PLASTIC SURGERY
UNIVERSITY OF MICHIGAN
ANN ARBOR, MI 48109

WU, WEN-CHIH, MD *
SECTION CHIEF, CARDIOLOGY
PROVIDENCE VA MEDICAL CENTER
ASSOCIATE PROFESSOR
DEPARTMENT OF MEDICINE
BROWN UNIVERSITY MEDICAL SCHOOL
PROVIDENCE, RI 02908

SCIENTIFIC REVIEW OFFICER

PLOUZEK, CATHIE, PHD
OFFICE OF RESEARCH AND DEVELOPMENT
VETERANS HEALTH ADMINISTRATION
DEPARTMENT OF VETERANS AFFAIRS
WASHINGTON , DC 20420

* Temporary Member. For grant applications, temporary members may participate in the entire meeting or may review only selected applications as needed.

Consultants are required to absent themselves from the room during the review of any application if their presence would constitute or appear to constitute a conflict of interest.
